# Differential contribution of mismatch repair genes in the processing of DNA damage

**DOI:** 10.1186/1750-9378-6-S1-A6

**Published:** 2011-08-11

**Authors:** Hernan Flores-Rozas

**Affiliations:** 1 College of Pharmacy and Pharmaceutical Sciences, Florida A&M University, Tallahassee, Florida, USA

## Background

Defects in mismatch repair (MMR) have been extensively documented as the underlying cause of several cancers, most notably colon cancer. Recently, MMR has been proposed to play a role in the transition to hormone independence in prostate cancer. It is proposed that MMR mediates the cytotoxic effects of DNA damaging agents by exerting a futile repair pathway which leads to double strand breaks (DSBs). Previous reports indicate that the sensitivity of cells defective in homologous recombination (HR) to the DNA alkylation is reduced by defects in MMR genes. The involvement of MMR in the pathogenesis of prostate cancer has only recently been investigated. Genetic differences in MMR genes between ethnic populations may account for different outcomes after therapy. These genetic backgrounds should be investigated further.

## Methods

We have assessed the contribution of different MMR genes to the processing of alkylation damage *in vivo*. We have directly visualized recombination complexes formed upon DNA damage using fluorescent protein (FP) fusions in cells containing different MMR backgrounds.

## Results

We find that *msh6* mutants are more resistant than wild type cells to MNNG, and that an *msh6* mutation rescues the sensitivity of *rad52* strains more efficiently than an *msh3* mutation. Analysis of RAD52-GFP tagged strains indicate that MNNG increases repair foci formation, and that the inactivation of the *MHS2* and *MSH6* genes but not *MSH3* gene result in a reduction of the number of foci formed. In addition, in the absence of HR, NHEJ could process the MNNG-induced DSBs as indicated by the formation of NHEJ-GFP tagged foci.

## Conclusions

These data suggest that processing of the alkylation damage by MMR, mainly by MSH2-MSH6, is essential for damage processing and MMR status should be evaluated prior to therapy.

